# Challenges in Reproducibility, Replicability, and Comparability of Computational Models and Tools for Neuronal and Glial Networks, Cells, and Subcellular Structures

**DOI:** 10.3389/fninf.2018.00020

**Published:** 2018-05-01

**Authors:** Tiina Manninen, Jugoslava Aćimović, Riikka Havela, Heidi Teppola, Marja-Leena Linne

**Affiliations:** ^1^Computational Neuroscience Group, BioMediTech Institute and Faculty of Biomedical Sciences and Engineering, Tampere University of Technology, Tampere, Finland; ^2^Laboratory of Signal Processing, Tampere University of Technology, Tampere, Finland

**Keywords:** astrocyte, computational model, glial cell, neuron, neuronal network, replicability, reproducibility, subcellular structure

## Abstract

The possibility to replicate and reproduce published research results is one of the biggest challenges in all areas of science. In computational neuroscience, there are thousands of models available. However, it is rarely possible to reimplement the models based on the information in the original publication, let alone rerun the models just because the model implementations have not been made publicly available. We evaluate and discuss the comparability of a versatile choice of simulation tools: tools for biochemical reactions and spiking neuronal networks, and relatively new tools for growth in cell cultures. The replicability and reproducibility issues are considered for computational models that are equally diverse, including the models for intracellular signal transduction of neurons and glial cells, in addition to single glial cells, neuron-glia interactions, and selected examples of spiking neuronal networks. We also address the comparability of the simulation results with one another to comprehend if the studied models can be used to answer similar research questions. In addition to presenting the challenges in reproducibility and replicability of published results in computational neuroscience, we highlight the need for developing recommendations and good practices for publishing simulation tools and computational models. Model validation and flexible model description must be an integral part of the tool used to simulate and develop computational models. Constant improvement on experimental techniques and recording protocols leads to increasing knowledge about the biophysical mechanisms in neural systems. This poses new challenges for computational neuroscience: extended or completely new computational methods and models may be required. Careful evaluation and categorization of the existing models and tools provide a foundation for these future needs, for constructing multiscale models or extending the models to incorporate additional or more detailed biophysical mechanisms. Improving the quality of publications in computational neuroscience, enabling progressive building of advanced computational models and tools, can be achieved only through adopting publishing standards which underline replicability and reproducibility of research results.

## 1. Introduction

All areas of science are facing problems with reproducibility and replicability (Baker, 2016; Eglen et al., 2017; Munafò et al., 2017; Rougier et al., 2017), and computational neuroscience is no exception. We aim to contribute to the ongoing discussion on reproducibility and replicability by presenting several efforts to systematize and compare, rerun and replicate, as well as reimplement, simulate, and reproduce models and knowledge within our fields of expertise. By comparability, we mean comparing either the simulation results of different simulation tools when the same model has been implemented in them or the simulation results of different models. By replicability, we mean rerunning the publicly available code developed by the authors of the study and replicating the original results from the study. By reproducibility, we mean reimplementing the model using the knowledge from the original study, often in a different simulation tool or programming language from the one reported in the study, and simulating it to verify the results from the study. These definitions are consistent with the terminology on replicability and reproducibility used in the literature (see also Crook et al., 2013; McDougal et al., 2016). However, there is an ongoing discussion on optimal use of terminology and several alternatives have been proposed (see e.g., Goodman et al., 2016; Rougier et al., 2017; Benureau and Rougier, 2018). The lack of universally accepted terminology, solutions proposed in other scientific disciplines, and possible solutions for computational neuroscience are also discussed by Plesser (2018). In order to focus on our findings and conclusions rather than terminology, we will adopt the definitions described above without further discussion about the alternatives.

There is an evident need to evaluate, compare, and systematize tools and models. With the increasing number of published models, it is becoming difficult to evaluate the unique contribution in each of them or assess the scientific rigor. The published articles might provide incomplete information, due to accidental mistakes or limited space and publication format. The original model implementations are not always available. The overhead of model reimplementation and reproduction of the results could be significant. More systematic model description (Nordlie et al., 2009), publishing the code in addition to the article (Eglen et al., 2017; Rougier et al., 2017), and efforts on independent reproduction and replication of the published models (Manninen et al., 2017; Rougier et al., 2017) improve quality and reliability of results in computational neuroscience. Better systematization and classification of the models provide more straightforward recommendations for the scientists initiating new projects or for the training of young researchers entering the field (see also Akil et al., 2016; Amunts et al., 2016; Nishi et al., 2016). All these can better support the reuse and extension of the published models which is often necessary when building models of complex phenomena. The development of new experimental techniques and the new experimental findings also pose new questions for the computational neuroscience. The models that address these questions are often built on top of the existing ones, and heavily depend on the reusability and reliability of the published work. These issues become even more important with the increasing interest and current intensive development of multiscale models. Multiscale models include fine details of all levels of physical organization (molecular reaction networks, individual cells, local neuronal networks, glial networks, large-scale networks, and even complete functional brain systems), and naturally such complex and demanding models must rely even more on the existing knowledge and models. Furthermore, as the complexity of the models increases it becomes more difficult to duplicate the original work even if only one parameter value is mistyped, or completely omitted.

The listed challenges have been extensively discussed within the computational neuroscience and neuroinformatics community. Several publications have proposed improvements in model development and description recommending a clear and compact tabular format for model description (see e.g., Nordlie et al., 2009; Topalidou et al., 2015; Manninen et al., 2017). The issue of reproducibility in computational neuroscience has been emphasized through development of model description and simulation tools: the standardization of tools significantly accelerates development of new models and reproduction of the published models. An overview of the existing simulation tools, their features and strengths, as well as a discussion on future developments are presented in recent publications (Brette et al., 2007; Crook et al., 2013; McDougal et al., 2016). In addition, journals rarely explicitly state that they accept replicability and reproducibility studies (Yeung, 2017). However, the ReScience initiative was started to encourage researchers to reimplement published models and reproduce the research results (Rougier et al., 2017). During the review process in the ReScience Journal, both the manuscript and the reimplementation of the model are tested and checked by the reviewers, and both are made publicly available for the scientific community. In our previous study (Manninen et al., 2017), we addressed the reproducibility of a small set of computational glial models. Based on this study, we emphasize the necessity for giving out all information about the models, such as the inputs, equations, parameters, and initial conditions, in a tabular format, in addition to making the model code publicly available. Similar holds for complex network-level models composed of many interacting neurons where every small error might lead to a large deviation in the simulation outcome.

Equally important concept that should be discussed in the context of reproducibility and replicability is the development of validation strategies for comparability of various computational models. Better mathematical and computational tools are needed to provide easy and user-friendly evaluation and comparison. As can be seen from the reviews of previous modeling work in the field (Manninen et al., 2010, 2018a,b), many new models are built on top of pre-existing models, with some further parameter estimation based on experimental data. Often the validation against existing similar models is too tedious to do and, consequently, is skipped. To facilitate the usability of models, future computational neuroscience research should pay more attention to the questions of reproducibility, replicability, and validation of simulation results. As indicated, this issue becomes even more important with the current trends toward developing multiscale models.

In this study, we evaluate a number of computational models describing a very diverse set of neural systems and phenomena, as well as simulation tools dedicated to these models. We evaluate and discuss a versatile choice of simulation tools, from simulation tools of biochemical reactions, to relatively new simulation tools of growth in cell cultures, to relatively mature and widely adopted tools for modeling spiking neuronal networks. The computational models are equally diverse, including the models of intracellular signal transduction for neurons and glial cells, in addition to single glial cells, neuron-glia interactions, and neuronal networks. Although we take into account a range of models, some classes of models are not considered in this study. We omit single neuron models which are already well developed, compared, and systematized in the literature (Izhikevich, 2004; Sterratt et al., 2011). Furthermore, we do not intend to provide any extensive evaluation of neuronal network models, which are numerous in the literature, but instead discuss an illustrative data-driven modeling example and specific reproducibility, replicability, and comparability issues that emerge in this type of studies. The models for glial networks and the larger models of neuron-glia networks are also excluded from this work and might be subject of future studies. Through the evaluation of examples under consideration, we present the state-of-the-art in reproducibility and replicability of computational models of neuronal and glial systems, summarize our recent findings about reproducibility in computational neuroscience, and propose some good practices based on our present and previous work.

## 2. Material and methods

### 2.1. Simulation tools

In this section, we describe a range of simulation tools utilized to simulate the spiking neuronal networks, biochemical reactions, and neuronal growth. Simulation tools that allow constructing, simulating, and analyzing whole-cell and neuronal circuit models attracted the most attention in the past and are among the most developed tools used in computational neuroscience. Typical models range from multicompartmental neurons integrating some level of morphological and physiological details of the certain neuron type to the highly abstract models containing large number of low-dimensional model neurons and statistical description of connectivity rules. In computational systems biology, the simulation tools developed for different kinds of biological systems, such as gene regulatory networks, metabolic networks, and signal transduction, have been the focus of development. These tools are relatively mature, standardized, and well-known in the research community. On the other hand, the simulation tools for neurodevelopment are not so well-known, and thus we give more details about these tools in the upcoming sections. All the simulation tools tested and compared in this work are listed in Table 1.

**Table 1:** List of simulation tools and model repositories.

**Tool/Repository**	**Website**	**References**
**SIMULATION TOOL**		
Brian	http://brian2.readthedocs.io/en/stable/index.html	Goodman and Brette, 2008
Copasi	http://copasi.org/	Hoops et al., 2006
Cortex3D	https://www.ini.uzh.ch/~amw/seco/cx3d/	Zubler and Douglas, 2009
Dizzy	http://magnet.systemsbiology.net/software/Dizzy/	Ramsey et al., 2005
GENESIS/ Kinetikit	http://genesis-sim.org/, https://www.ncbs.res.in/faculty/bhalla-kinetikit	Wilson et al., 1989; Bower and Beeman, 1998; Bhalla and Iyengar, 1999; Bhalla, 2001, 2002
Gepasi	http://www.gepasi.org/	Mendes, 1993, 1997; Mendes and Kell, 1998
Jarnac/JDesigner	http://jdesigner.sourceforge.net/	Sauro, 2000, 2001
Narrator	https://omictools.com/narrator-tool	Mandel et al., 2007
NEST	http://www.nest-simulator.org/	Eppler et al., 2015
NETMORPH	http://www.netmorph.org/Home, http://www.scholarpedia.org/article/NETMORPH	Koene et al., 2009
NEURON	https://www.neuron.yale.edu/neuron/	Carnevale and Hines, 2006
PyNN	http://neuralensemble.org/PyNN/	Davison et al., 2009
SimTool	Request from the author	Aho, 2003
Systems Biology Toolbox	http://www.sbtoolbox.org/	Schmidt and Jirstrand, 2006
XPPAUT	http://www.math.pitt.edu/~bard/xpp/xpp.html	Ermentrout, 2002
**MODEL REPOSITORY**		
DOQCS	http://doqcs.ncbs.res.in/	Sivakumaran et al., 2003
DRYAD	http://datadryad.org/	
ModelDB	http://senselab.med.yale.edu/modeldb/	Migliore et al., 2003; Hines et al., 2004

*This table lists the names of the simulation tools and model repositories as well as their websites and references*.

#### 2.1.1. Simulation tools for biochemical reactions

Mathematical modeling of biochemistry is important for understanding complex biochemical processes that underlie many neuronal, glial, and synaptic phenomena. Recent interest in modeling biochemical networks in systems biology and in neuroscience have provided several tools that can be used to simulate time-series behavior of the networks (see e.g., Lemerle et al., 2005; Pettinen et al., 2005; Alves et al., 2006; Gilbert et al., 2006; Strömbäck et al., 2006; Wierling et al., 2007; Bergmann and Sauro, 2008; Blackwell, 2013; Schöneberg et al., 2014; Bartocci and Lió, 2016; Olivier et al., 2016). In this study, we used the following simulation tools: GENESIS/Kinetikit (Wilson et al., 1989; Bower and Beeman, 1998; Bhalla and Iyengar, 1999; Bhalla, 2001, 2002), Gepasi (Mendes, 1993, 1997; Mendes and Kell, 1998), Jarnac/JDesigner (Sauro, 2000, 2001), XPPAUT (Ermentrout, 2002), SimTool (Aho, 2003), Dizzy (Ramsey et al., 2005), Copasi (Hoops et al., 2006), NEURON (Carnevale and Hines, 2006), Systems Biology Toolbox (Schmidt and Jirstrand, 2006), and Narrator (Mandel et al., 2007) (see Table 1). Here, we do not provide any detailed overview of the simulation tools, because the topic has been already extensively discussed previously. However, we want to point out the differences in these tools by providing information about the methods used for modeling and simulation.

In the listed simulation tools, the model is often implemented using chemical reactions presented by the law of mass action and Michaelis-Menten kinetics. These reactions form coupled ordinary differential equations (ODEs) presenting the biochemical network, and these equations are then solved numerically when simulating the model. However, for example, in XPPAUT, the model is directly implemented using the ODEs and not the chemical reactions. Several of the tools also provide stochastic approaches to model and simulate the reactions (see e.g., Manninen et al., 2006a; Gillespie, 2007), such as the discrete-state Gillespie stochastic simulation algorithm (Gillespie, 1976, 1977) and τ-leap method (Gillespie, 2001), as well as continuous-state chemical Langevin equation (Gillespie, 2000) and several other stochastic differential equations (SDEs, Manninen et al., 2006a,b). Few simulation tools providing hybrid approaches also exist. They combine either deterministic and stochastic methods or different stochastic methods (see e.g., Salis et al., 2006; Lecca et al., 2017). The increased computing power has recently made it possible also to take into account diffusion processes. The reaction-diffusion simulation tools often use combined Gillespie algorithm or τ-leap method for both reaction and diffusion processes, such as STEPS (Wils and De Schutter, 2009; Hepburn et al., 2012) and NeuroRD (Oliveira et al., 2010), or track each molecule individually in a certain volume with Brownian dynamics combined with a Monte Carlo procedure for reaction events, such as MCell (Stiles and Bartol, 2001; Kerr et al., 2008) and Smoldyn (Andrews et al., 2010). Few studies to compare different reaction-diffusion tools exist (Dobrzyński et al., 2007; Oliveira et al., 2010; Schöneberg et al., 2014). In this study, however, we were only interested in comparing simulation tools with simple reaction models, and thus reaction-diffusion tools and models were not tested. The more detailed testing of reaction-diffusion tools remains for future work and will most probably be accelerated once more models for reaction-diffusion systems become available. The simulation tools for biochemical reactions addressed in this work have been studied in detail in our previous work (Pettinen et al., 2005; Manninen et al., 2006c; Mäkiraatikka et al., 2007) and the here presented results are a summary of our previous work. We recommend to consult the earlier studies for more details.

#### 2.1.2. Simulation tools for neurodevelopment

We examined relatively new and promising tools for modeling neurodevelopment. They facilitate exploring through computational means individual biophysical mechanisms involved in development and growth of neuronal circuits and analyzing the properties that arise from those mechanisms. We examined two simulation tools, NETMORPH (Koene et al., 2009) and Cortex3D (Zubler and Douglas, 2009), the full references and links to these tools are given in Table 1. Because these tools are newer, less known and used than the other simulation tools presented in this study, we describe them with additional details.

NETMORPH implements a phenomenological model of neurite growth (in 2D or 3D) based on extensive statistical characterization of dendritic morphology in developing circuits conducted by the authors of the simulation tool (van Pelt and Uylings, 2003; Koene et al., 2009). It can simulate formation of synaptic contacts based on morphology and the proximity between axonal and dendritic segments. The simulation tool was developed in C++ under Unix/Linux and can be installed straightforwardly under the same environment. In Windows, it can be installed using Cygwin. The inputs are text files containing a list of model components and the belonging parameters. The components include description of neuronal population, morphology for each neuron type, parameters determining synapse formation, and a set of flags describing the format of simulation outputs. Model equations are evaluated at fixed time steps, and the output can be generated either at specified time points or at the end of a simulation. Three types of outputs are possible: visualization of neuronal morphologies and networks, raw data containing the list of all generated model components, and the statistics computed from the raw data.

Cortex3D is a simulation platform that supports modeling biophysical and mechanistic details of neural development. As such it does not specify any particular model but rather a set of underlying mechanisms that can be used to implement and simulate user-defined models. The mechanisms embedded into the simulation tool include discretization of space occupied by a model, production, diffusion, degradation, and reactions between chemical species, the effect of mechanical and chemical forces between components of the model, and movement of objects inside the model. The model is solved at a fixed time grid. Parts of the model represented by dynamical equations are solved using Euler method, but numerical integration is replaced by analytical solution whenever possible to avoid overshoot for large time steps. The simulation tool is organized into layers of abstraction, with the discretization of space mapped into the lowest layer, the physical properties of the objects being specified one layer above, and the biological properties mapped into the top two layers. Most of the user-defined model properties can be specified in the top “cell” layer (Zubler and Douglas, 2009). The simulation tool was implemented in Java and is easy to install on any platform. A parallelized version of the simulation tool is also available (Zubler et al., 2011, 2013). Recently, a new simulation tool capable of modeling neuronal tissue development, inspired by Cortex3D and based on the same computational principles, was proposed (https://biodynamo.web.cern.ch/). To specify a model in Cortex3D, a user should write Java module containing the description of model components, interactions between the components, and model parameters. The output of the simulation tool is an Extensible Markup Language (XML) schema compatible with NeuroML containing the details of the obtained model. The simulation tool also integrates Java packages that allow visualization of the simulation evolution. We tested and compared NETMORPH and Cortex3D by implementing and running the same model compatible with both tools and evaluating the simulated data. In addition to tool evaluation, we were interested in promoting the usefulness of these new and underutilized simulation tools.

#### 2.1.3. Simulation tools for neuronal networks

Computational studies of individual neurons and neuronal circuits were the first attempts at computational modeling in neuroscience, have the longest history, and are still the most represented level of abstraction when addressing the function and organization of neuronal systems. They originate from the experimentally verified models of neurons, the ground truth of neuron electrophysiology based on Hodgkin-Huxley (HH, Hodgkin and Huxley, 1952) formalism and the mathematical description of ion channel dynamics. Individual neurons can be described either as single compartmental models representing the somatic membrane potential or as multicompartmental models including parts of dendritic and axonal arbors. In addition, the simulation tools provide a number of simpler and computationally less demanding neuron models based on an integrate-and-fire (IF) modeling formalism. They also provide mechanisms to construct networks of model neurons, from generic random networks to specific brain circuits. Some of these simulation tools also have a capacity to implement subcellular models (NEURON, XPPAUT, and GENESIS). Consequently, these simulation tools are widely accepted and well known within the scientific community and can be considered mature. All of these simulation tools implement deterministic methods to solve the systems of ODEs, and some of them also have possibility to implement SDE models (see e.g., Stimberg et al., 2014). Deterministic integration methods for solving ODEs use either a fixed or adaptable integration step size. For some neurons of IF type, it is possible to solve the ODE exactly between the spike times and update the model at each spike time, thus significantly increasing the accuracy of numerical integration. The extensive discussion about numerical methods can be found in the literature (Rotter and Diesmann, 1999; Lytton and Hines, 2005; Carnevale and Hines, 2006; Brette et al., 2007; Stimberg et al., 2014). Here, we do not aim at giving an overview of simulation tools or comparing their properties, these topics have been extensively discussed elsewhere (see e.g., Brette et al., 2007; McDougal et al., 2016). Instead, we will present our unpublished user experiences from describing and simulating spiking neuronal networks using NEST (Eppler et al., 2015), Brian (Goodman and Brette, 2008; Stimberg et al., 2014), and PyNN (Davison et al., 2009).

### 2.2. Models

In this section, we give an overview of computational models used in our reproducibility, replicability, and comparability studies. These include the models of intracellular signal transduction for neurons and glial cells, in addition to single glial cells, neuron-glia interactions, and neuronal networks. We tabulated the following properties for the models whenever suitable:

- **Neuron model:** Multicompartmental or point neuron models adopted from the literature.
- **Synapse model:** Types of synaptic models and receptors.
- **Neuron-astrocyte synapse model:** Types of synaptic models and receptors.
- **Connectivity:** Statistical description of connectivity schemes.
- **Intracellular signaling;** Intracellular calcium signaling (e.g., leaks, pumps, and receptors that are not named under other categories) in addition to different intracellular chemical species taken into account either in neurons or astrocytes.
- **Data analysis:** Description of the methods used to analyze *in silico* data from spiking neuronal network models.

Some of the models we used in this study were found available in model repositories. These repositories are listed in Table 1.

#### 2.2.1. Neuronal signal transduction models

More than a hundred intracellular biochemical species are important in synaptic plasticity. Hundreds of neuronal signal transduction models have been developed to test the criticality of different chemical species. Several reviews of the models exist, some focus on just a few different models whereas others give an overview of more than hundred models (Brown et al., 1990; Neher, 1998; Hudmon and Schulman, 2002a,b; Bi and Rubin, 2005; Holmes, 2005; Wörgötter and Porr, 2005; Ajay and Bhalla, 2006; Klipp and Liebermeister, 2006; Zou and Destexhe, 2007; Morrison et al., 2008; Ogasawara et al., 2008; Bhalla, 2009; Ogasawara and Kawato, 2009; Tanaka and Augustine, 2009; Urakubo et al., 2009; Castellani and Zironi, 2010; Gerkin et al., 2010; Graupner and Brunel, 2010; Hellgren Kotaleski and Blackwell, 2010; Manninen et al., 2010; Shouval et al., 2010). The models range from a simple models with just a single reversible reaction to very detailed models with several hundred reactions. In Table 2, we list the neuronal signal transduction models for plasticity that we evaluated in this study. The models by d'Alcantara et al. (2003) and Delord et al. (2007) were the simplest with just a few reactions, whereas the model by Zachariou et al. (2013) had both pre- and postsynaptic single-compartmental neurons and rest of the models had very detailed intracellular signaling pathways taken into account. The models by d'Alcantara et al. (2003), Delord et al. (2007), and Zachariou et al. (2013) we used in the reproducibility studies. In the comparability studies (Manninen and Linne, 2008; Manninen et al., 2011), we tested the models by d'Alcantara et al. (2003), Hayer and Bhalla (2005), Lindskog et al. (2006), Delord et al. (2007), Nakano et al. (2010), and Kim et al. (2010).

**Table 2:** Summary of the neuronal signal transduction models.

**Model**	**Neuron model**	**Synapse model**	**Intracellular signaling**
d'Alcantara et al., 2003	No	AMPAR	CaM, CaMKII, CaN, DARPP32 or I1, PP1
Hayer and Bhalla, 2005	No	AMPAR, NMDAR	AC1, AC2, AMP, Ca^2+^, CaM, CaMKII, cAMP, CaN, I1, Ng, PDE1, PKA, PKC, PP1, PP2A
Lindskog et al., 2006	No	D_1_R	AC5, AMP, ATP, CaM, CaMKII, cAMP, CaN, Cd5k, DARPP32, G protein, PDE1, PDE4, PKA, PP1, PP2A
Delord et al., 2007	No	No	Kinase, phosphatase, substrate
Nakano et al., 2010	No	AMPAR, D_1_R	AC5, AMP, ATP, Ca^2+^, CaM, CaMKII, cAMP, CaN, Cd5k, CK1, DARPP32, G protein, I1, PDE1, PDE2, PKA, PP1, PP2A, PP2C
Kim et al., 2010	No	D_1_R	AC1, AC8, AMP, ATP, Ca^2+^, CaM, CaMKII, cAMP, CaN, G protein, I1, PDE1B, PDE4, PKA, PP1
Zachariou et al., 2013	Presyn.: HH (Kdr, Na, N-type VGCC), postsyn.: HH (Kdr, L-type VGCC, Na)	Presyn.: CB_1_, postsyn.: AMPAR, GABA_A_R	Postsyn.: 2-AG, Ca^2+^ (Ca^2+^ leak from ER into cyt, Ca^2+^ leak from ext into cyt, PMCA, SERCA), ${\text{Ca}}_{\text{ER}}^{2+}$, DAG

***Neuron model:** pre- and postsynaptic point neuron models. **Synapse model:** pre- and postsynaptic receptors. **Intracellular signaling:** intracellular calcium signaling (e.g., leaks and pumps that are not named under other categories) in addition to different intracellular chemical species in pre- and postsynaptic neurons. 2-AG, 2-arachidonoylglycerol; AC1, adenylyl cyclase type 1; AC2, AC type 2; AC5, AC type 5; AC8, AC type 8; AMP, adenosine monophosphate; AMPAR, α-amino-3-hydroxy-5-methyl-4-isoxazolepropionic acid receptor; ATP, adenosine triphosphate; Ca^2+^, calcium ion; CaM, calmodulin; CaMKII, Ca^2+^/CaM-dependent protein kinase II; cAMP, cyclic AMP; CaN, calcineurin; CB_1_, cannabinoid type 1 receptor; Cdk5, cyclin-dependent kinase 5; CK1, casein kinase 1; cyt, cytosol; D_1_R, dopamine receptor; DAG, diacylglycerol; DARPP32, dopamine- and cAMP-regulated neuronal phosphoprotein of 32 kDa; ER, endoplasmic reticulum; ext, extracellular space; GABA_A_R, gamma-aminobutyric acid type A receptor; HH, Hodgkin-Huxley; I1, inhibitor 1; Kdr, delayed rectifier potassium current; Na, sodium current; Ng, neurogranin; NMDAR, N-methyl-D-aspartate receptor; PDE1, phosphodiesterase type 1; PDE1B, PDE type 1B; PDE2, PDE type 2; PDE4, PDE type 4; PKA, cAMP-dependent protein kinase; PKC, protein kinase C; PMCA, plasma membrane Ca^2+^ ATPase; PP1, protein phosphatase 1; PP2A, PP type 2A; PP2C, PP type 2C; SERCA, sarco/endoplasmic reticulum Ca^2+^-ATPase; VGCC, voltage-gated Ca^2+^ channel*.

#### 2.2.2. Astrocyte models

Similarly to neuronal signal transduction models, hundreds of single astrocyte, astrocytic network, and neuron-astrocyte interaction models have been developed to study different phenomena. Several reviews of computational astrocyte and neuron-astrocyte models have appeared during the last few years, some focusing only to a few models and some giving a general overview of the field (see e.g., Jolivet et al., 2010; Mangia et al., 2011; De Pittà et al., 2012, 2016; Fellin et al., 2012; Min et al., 2012; Volman et al., 2012; Wade et al., 2013; Linne and Jalonen, 2014; Tewari and Parpura, 2014; Manninen et al., 2018a,b). In Table 3, we list the models that we evaluated in this study. We chose five single astrocyte and signal transduction models (Di Garbo et al., 2007; Lavrentovich and Hemkin, 2008; De Pittà et al., 2009; Dupont et al., 2011; Riera et al., 2011a,b) and four neuron-astrocyte interaction models (Nadkarni and Jung, 2003; Silchenko and Tass, 2008; Wade et al., 2011, 2012). Silchenko and Tass (2008) used a two-compartmental neuron model, whereas the other three (Nadkarni and Jung, 2003; Wade et al., 2011, 2012) used single-compartmental models. The models by Nadkarni and Jung (2003), Di Garbo et al. (2007), Silchenko and Tass (2008), Lavrentovich and Hemkin (2008), De Pittà et al. (2009), Riera et al. (2011a,b), Dupont et al. (2011), and Wade et al. (2011, 2012) were tested in the reproducibility studies (see also Manninen et al., 2017, 2018b). In addition, the models by Lavrentovich and Hemkin (2008), De Pittà et al. (2009), Riera et al. (2011a,b), and our modified version of the model by Dupont et al. (2011) were used in the comparability study (see also Manninen et al., 2017).

**Table 3:** Summary of the astrocyte and neuron-astrocyte models.

**Model**	**Neuron model**	**Neuron-astrocyte synapse model**	**Intracellular signaling in neuron**	**Intracellular signaling in astrocyte**
Nadkarni and Jung, 2003	Postsyn.: HH (Kdr, Na)	Postsyn. voltage ↦ astro IP_3_,astro Ca^2+^ ↦ postsyn. current	No	Ca^2+^ (CICR via IP_3_R, Ca^2+^ leak from ER into cyt, SERCA), IP_3_, active fraction of IP_3_R
Di Garbo et al., 2007	No	Astro: P2XR, P2YR	No	Ca^2+^ (CCE, CICR via IP_3_R, Ca^2+^ efflux, Ca^2+^ leak from ER into cyt, Ca^2+^ leak from ext into cyt, SERCA), ${\text{Ca}}_{\text{ER}}^{2+}$, IP_3_, active fraction of IP_3_R
Silchenko and Tass, 2008	Postsyn.: Pinsky-Rinzel,HH (AHP, Kdr, L-type VGCC, Na)	Postsyn.: AMPAR, NMDAR, astro: mGluR	Ca^2+^	Ca^2+^ (CICR via IP_3_R, Ca^2+^ efflux, glutamate-dependent Ca^2+^ influx, Ca^2+^ influx, Ca^2+^ leak from ER into cyt, SERCA), ${\text{Ca}}_{\text{ER}}^{2+}$, IP_3_, vesicle cycle, glutamate release
Lavrentovich and Hemkin, 2008	No	No	No	Ca^2+^ (CICR via IP_3_R, Ca^2+^ efflux, Ca^2+^ influx, Ca^2+^ leak from ER into cyt, SERCA), ${\text{Ca}}_{\text{ER}}^{2+}$, IP_3_
De Pittà et al., 2009	No	No	No	Ca^2+^ (CICR via IP_3_R, Ca^2+^ leak from ER into cyt, SERCA), IP_3_, active fraction of IP_3_R
Riera et al., 2011a,b	No	No	No	Ca^2+^ (CCE, CICR via IP_3_R, Ca^2+^ efflux, Ca^2+^ influx via channels, Ca^2+^ leak from ER into cyt, SERCA), ${\text{Ca}}_{\text{free}}^{2+}$, IP_3_, active fraction of IP_3_R
Dupont et al., 2011	No	Astro: mGluR	No	Ca^2+^ (CICR via IP_3_R, Ca^2+^ efflux, Ca^2+^ influx, Ca^2+^ leak from ER into cyt, SERCA), DAG, IP_3_, fraction of Ca^2+^-inhibited IP_3_R, active fraction of PKC
Wade et al., 2011	Postsyn.: LIF	Tsodyks ↦ astro IP_3_ and syn. current, astro Ca^2+^ ↦ postsyn. NMDAR,astro glutamate ↦ Tsodyks	No	Ca^2+^ (CICR via IP_3_R, Ca^2+^ leak from ER into cyt, SERCA), IP_3_, active fraction of IP_3_R, glutamate release
Wade et al., 2012	Postsyn.: LIF	Postsyn. 2-AG ↦ astro IP_3_, astro glutamate ↦ syn. current	Postsyn.: 2-AG, depression, potentiation	Ca^2+^ (CICR via IP_3_R, Ca^2+^ leak from ER into cyt, SERCA), IP_3_, active fraction of IP_3_R, glutamate release

***Neuron model:** postsynaptic multicompartmental and point neuron models. **Neuron-astrocyte synapse model:** Tsodyks-Pawelzik-Markram model; postsynaptic and astrocytic receptors. **Intracellular signaling in neuron:** intracellular chemical species in postsynaptic neuron. **Intracellular signaling in astrocyte:** intracellular calcium signaling (e.g., leaks, pumps, and receptors that are not named under other categories) in addition to different intracellular chemical species in astrocyte. We only implemented the astrocyte component of the model by Di Garbo et al. (2007), and not the neuron component at all. 2-AG, 2-arachidonoylglycerol; AHP, after-hyperpolarization current; AMPAR, α-amino-3-hydroxy-5-methyl-4-isoxazolepropionic acid receptor; ATP, adenosine triphosphate; Ca^2+^, calcium ion; CCE, capacitive Ca^2+^ entry; CICR, Ca^2+^-induced Ca^2+^ release; cyt, cytosol; DAG, diacylglycerol; ER, endoplasmic reticulum; ext, extracellular space; HH, Hodgkin-Huxley; IP_3_, inositol trisphosphate; IP_3_R, IP_3_ receptor; Kdr, delayed rectifier potassium current; LIF, leaky integrate-and-fire; mGluR, metabotropic glutamate receptor; Na, sodium current; NMDAR, N-methyl-D-aspartate receptor; P2XR, ionotropic purinergic ATP receptor; P2YR, purinergic G-protein-coupled metabotropic receptor; PKC, protein kinase C; SERCA, sarco/ER Ca^2+^-ATPase; VGCC, voltage-gated Ca^2+^ channel*.

#### 2.2.3. Spiking neuronal network models

Spiking neuronal network models are numerous in the literature and used to model various phenomena and brain structures. In order to constrain this evaluation to a reasonable set of models, we selected only those models which are developed for the spontaneously synchronized population activity from dissociated neuronal cultures *in vitro* (for more details, see Robinson et al., 1993; Teppola et al., 2011). The focus on data-driven models gives us an opportunity to emphasize the need for reproduction of both model and data analysis tools. We compared several publications (Latham et al., 2000; Giugliano et al., 2004; French and Gruenstein, 2006; Gritsun et al., 2010, 2011; Baltz et al., 2011; Maheswaranathan et al., 2012; Mäki-Marttunen et al., 2013; Masquelier and Deco, 2013; Yamamoto et al., 2016; Lonardoni et al., 2017), that use similar models, address similar questions, and should converge toward similar conclusions. Some differences emerge from the experimental preparation, from the recording technology, or variations in model composition. The publications by Gritsun et al. (2010, 2011) present two parts of the same study. They are considered as one study, but are presented separately in Table 4 due to the small differences in model construction and data analysis. The publication by Mäki-Marttunen et al. (2013) is not, strictly speaking, modeling the experimental data but rather uses the theoretical concepts to explore models and synthetic data typical for this same type of experiments. All of the studies under consideration implement networks of point-neurons (a few hundred to few thousand neurons) with none or short-term plasticity in synapses and statistical description of connectivity. Similar models have been extensively analyzed in theoretical studies exploring feasible dynamical regimes, and some of them are available in public repositories dedicated to reproducible model development (see OpenSourceBrain; http://www.opensourcebrain.org/). In this study, we do not consider recent attempts to model the effects of non-neuronal cells, and we also leave out the mean field approaches to modeling the same type of experiments and data. The 10 selected studies are summarized in Table 4.

**Table 4:** Summary of the spiking neuronal network models.

**Model**	**Neuron model**	**Synapse model**	**Connectivity**	**Data analysis**
Latham et al., 2000	QIF/Theta, AHP, Ref, excitatory and inhibitory	exp-cond.	Nonstructured, distance-based	Burst detection: none; Measures: rasterplot, GFR
Giugliano et al., 2004	LIFa, excitatory	exp-curr.	Nonstructured	Burst detection: not given; Measures: burst structure, burst count/freq.
French and Gruenstein, 2006	LIF, AHP, Ref, T-type VGCC	alpha-curr., depression	SW	Burst detection: none; Measures: burst size (number of active neurons), speed of burst propagation
Gritsun et al., 2010	Izhikevich, excitatory and inhibitory	exp-curr., Tsodyks	Nonstructured	Burst detection: GFR; Measures: burst structure
Gritsun et al., 2011	Izhikevich, excitatory and inhibitory	exp-curr., Tsodyks	Nonstructured, intense neurons	Burst detection: ISI-cell.; Measures: burst count/freq.
Baltz et al., 2011	LIF, AHP, Ref, T-type VGCC, excitatory	AMPAR, NMDAR, Tsodyks	Nonstructured	Burst detection: ISI-cell.; Measures: rasterplots, GFR, burst structure, burst count/freq.
Maheswaranathan et al., 2012	Izhikevich, excitatory and inhibitory	exp	SW	Burst detection: GFR; Measures: rasterplots, GFR, burst structure, spectral analysis, PCA
Mäki-Marttunen et al., 2013	LIF, HH (Kdr, K-slow, Na, NaP), excitatory and inhibitory	(with LIF) exp-curr., Tsodyks;(with HH) AMPAR, NMDAR, GABA_A_R	Nonstructured, distance-based, SW, complex, simulated	Burst detection: ISI-pop.; Measures: rasterplots, burst structure, connectivity, graph measures
Masquelier and Deco, 2013	LIF, AHP, excitatory	AMPAR, NMDAR, Tsodyks	Nonstructured	Burst detection: GFR; Measures: burst count/freq.
Yamamoto et al., 2016	LIF, AHP, Ref, T-type VGCC, excitatory	biexp-cond.	Nonstructured	Burst detection: not clear; Measures: rasterplots, burst count/freq., connectivity
Lonardoni et al., 2017	AdExp, excitatory and inhibitory	biexp-cond., AMPAR, GABA_A_R, NMDAR, Tsodyks	Distance-based (alternatives considered)	Burst detection: GFR; Measures: burst structure, burst count/freq., GFR, connectivity, burst propagation, graph measures

***Neuron model:** point neuron model, one (excitatory) or two (excitatory and inhibitory) neuronal populations. **Synapse model:** exponential (exp.), bi-exponential (biexp.), or alpha postsynaptic current (curr.) or conductance (cond.); Tsodyks-Markram model; synaptic receptors. **Connectivity:** network connectivity, nonstructured (equal probability of connection for every pair of neurons), distance-based (probability of connection decreases with the distance between somata), small-world and other complex connectivity schemes, intense neurons (nonstructured, but a subset of neurons has particularly strong synapses), simulated (morphology-based connectivity simulated using NETMORPH). **Data analysis:** burst detection (identifying periods of global synchronization from the data), categories: from inter-spike-intervals of individual neurons (ISI-cell.), from inter-spike-intervals of the population (ISI-pop.), from global firing rates (GFR). Data measures: burst structure (length, number of spikes per burst etc.), burst count or frequency or statistics of inter-burst-intervals, frequency analysis, burst propagation through network, analysis of connectivity (physical or functional/from spike trains), graph measures of connectivity. AdExp, adaptive exponential; AHP, after-hyperpolarization current; AMPAR, α-amino-3-hydroxy-5-methyl-4-isoxazolepropionic acid receptor; GABA_A_R, gamma-aminobutyric acid type A receptor; HH, Hodgkin-Huxley; Kdr, delayed rectifier potassium current; K-slow, slow potassium current; LIF and LIFa, leaky integrate and fire without and with adaptation; Na, sodium current; NaP, persistent sodium current; NMDAR, N-methyl-D-aspartate receptor; PCA, principal component analysis; QIF, quadratic integrate-and-fire; Ref, refractory current; SW, small-world connectivity; Theta, theta model; T-type VGCC, T-type voltage-gated Ca^2+^ channel (in bursting neurons)*.

## 3. Results

We here evaluate first the simulation tools we used for biochemical reactions, growth in cell cultures, and spiking neuronal networks, and last the computational models for signal transduction in neurons, astrocytes, and spiking neuronal networks.

### 3.1. Evaluation of simulation tools

#### 3.1.1. Simulation tools for biochemical reactions

In our previous studies, we have extensively used and evaluated both deterministic and stochastic simulation tools for biochemical reactions (see Table 1), categorized their basic properties, benefits, and drawbacks, as well as tested the tools by implementing test cases and running simulations (Pettinen et al., 2005; Manninen et al., 2006c; Mäkiraatikka et al., 2007). At first, we tested four deterministic simulation tools, GENESIS/Kinetikit (versions 2.2 and 2.2.1 of the GENESIS and versions 8 and 9 of the Kinetikit), Jarnac/JDesigner (version 2.0 of Jarnac and version 1.8k of JDesigner), Gepasi (version 3.30), and SimTool, by implementing the same test case for every simulation tool and running simulations (Pettinen et al., 2005). Next, we tested three stochastic simulation tools, Dizzy (version 1.11.2), Copasi (release candidate 1, build 17), and Systems Biology Toolbox (version 1.5), the same way (Manninen et al., 2006c; Mäkiraatikka et al., 2007). Last, we tested the possibility to easily exchange models between stochastic simulation tools using Systems Biology Markup Language (SBML) (Mäkiraatikka et al., 2007). As a surprise, only a few of the tools that were supposed to support SBML import were capable of simulating the selected test case when imported as SBML file (Mäkiraatikka et al., 2007). Of the tools that we tested for that study, only Dizzy, Narrator, and XPPAUT succeeded in simulating the imported SBML file. We found out in all of our studies (Pettinen et al., 2005; Manninen et al., 2006c; Mäkiraatikka et al., 2007) that the simulation results between the tools were convergent. Using the same test case as by Pettinen et al. (2005), we also found out in a separate set of tests that NEURON produced similar results as the other tools mentioned above. Based on our studies, we concluded that the comparability of the simulation results needed several requirements to be fulfilled. First, the usability of the simulation tools and existence of proper manuals were crucial. For example, even beginners were able to use Dizzy, Gepasi, Copasi, and Jarnac/JDesigner, but former experience in MATLAB^Ⓡ^ was required for Systems Biology Toolbox and SimTool. Second, the lack of standards and interfaces between tools also made the comparability problematic. For example related to SBML import, graphical user interfaces designed to help the SBML import were not intuitive, the error messages were not informative enough, and not all the SBML levels were supported. Furthermore, for stochastic simulations with the Gillespie stochastic simulation algorithm, all the chemical reactions in the model had to be implemented as irreversible. Although the test case was implemented and exported with only irreversible reactions, we found simulation tools that mistook some of the irreversible reactions for reversible reactions during the SBML import and thus, we were not able to run stochastic simulations with these tools (Mäkiraatikka et al., 2007). In addition, problems arose when having various biochemical and physiological units because during manual conversion the chance of making errors was significant. Third, the utilization of realistic external stimuli was not possible in all simulation tools. Out of the tested simulation tools, GENESIS/Kinetikit was one of the good examples where external stimuli were enabled. Fourth, only a few of the tools had built-in automated parameter estimation methods to tune the models and their unknown parameter values. However, several methodology improvements have been made in the field in order to perform sophisticated parameter estimation. The use may, however, require some more detailed knowledge in computer science. Thus, all these difficulties and deficiencies present in simulation tools can make the comparison of simulation results difficult.

#### 3.1.2. Simulation tools for neurodevelopment

We tested two simulation tools dedicated to modeling neurodevelopmental mechanisms, NETMORPH and Cortex3D (Aćimović et al., 2011). While other simulation tools (e.g., NEURON) can be used to implement models at particular developmental age, NETMORPH and Cortex3D implement the mechanisms behind developmental changes. NETMORPH and Cortex3D can be used to address the same questions, but they are fundamentally different in methodology, approach, and philosophy of modeling being therefore complementary rather than competing. NETMORPH and Cortex3D were implemented using different programming languages. Running simulations beyond making simple changes to the provided examples required a deeper understanding of the tools.

**Figure 1:**
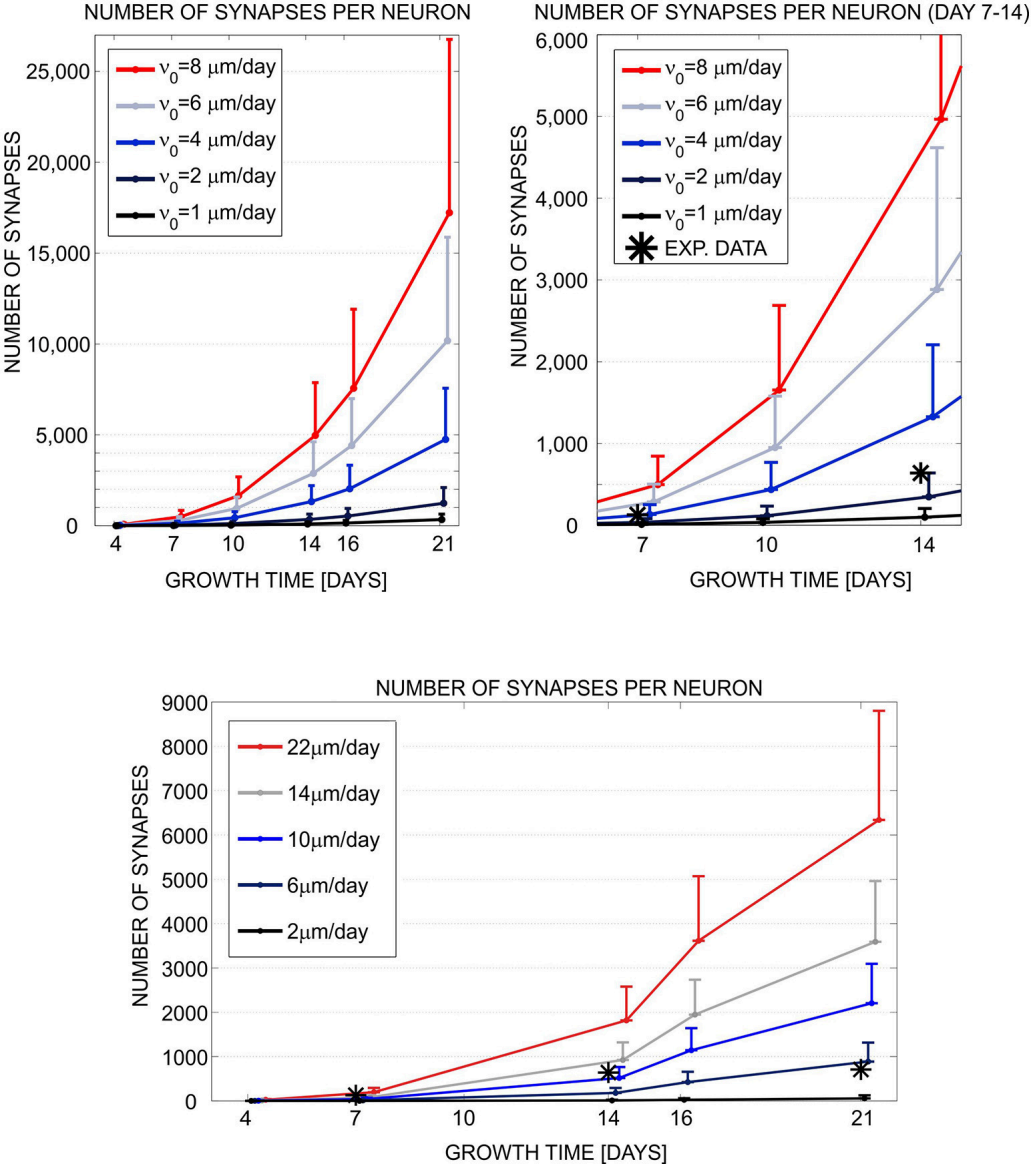
Evaluation and comparison of the neuronal growth simulation tools (NETMORPH and Cortex3D). Panels illustrate the increase in synapse counts during simulation time equivalent to 4–21 days *in vitro*. The number and position of somata were fixed. For each neuron, the neurites grew according to the implemented model and formed synaptic contacts based on proximity between axonal and dendritic branches. In this figure, we varied one of the parameters that controlled neurite growth, the elongation rate ν_0_ (see legend), and different colors correspond to different parameter values. The results show mean (line) and standard deviation (bar) for the number of synapses per neuron, averaged over all neurons in the culture. Stars indicate experimental values extracted from the literature (Ichikawa et al., 1993). **(Top left)** Synapse counts obtained from NETMORPH, elongation rates equal to 1, 2, 4, 6, and 8 μm/day. **(Top right)** Zoomed interval 7–14 days from the panel (Top left). **(Bottom)** Synapse counts obtained from Cortex3D, elongation rates equal to 2, 6, 10, 14, and 22 μm/day. x axis—growth time in days, y axis—number of synapses per neuron. For days 4–14 and ν_0_ = 2μm/day (NETMORPH) or ν_0_ = 10μm/day (Cortex3D), the simulated values corresponded to the experimental ones. After 14 days the simulated values increased while the experimental values saturated as no synaptic pruning was implemented in this test. The neurite growth was slower for Cortex3D which was visible from the values for ν_0_. Reproduced from Aćimović et al. (2011) with permission from Hindawi.

In short, we implemented a phenomenological model, compatible with both simulation tools, of neurite growth and formation of synaptic contacts based on morphology criteria (for more details see Aćimović et al., 2011). The choice is determined by model components and mechanisms available in NETMORPH. To analyze the simulation results, we wrote own MATLAB^Ⓡ^ code which converted both simulated data sets to the same format and computed the statistics from the data. We examined the simulated morphologies, analyzed the number of generated synapses at different simulation times, and compared the synapse counts to the experimental data extracted from the literature (see Figure 1). As a conclusion, the two simulation tools produced qualitatively similar growth dynamics. The simulated results were consistent with the experimental data in the early phase of growth but deviated in the latter phase. Cortex3D gave somewhat shorter neurites with less synaptic contacts and less precise control of the orientation of neurite segments than NETMORPH. While NETMORPH implements a set of equations derived to produce precise statistics for all relevant parameters of neurite morphology, Cortex3D focuses on the underlying mechanisms of growth, for example the tensions resulting from elongation and the production of resources needed for growth. These mechanisms affect neurite morphology in a complex and not fully predictable way. The computational model used for testing and comparing the simulation tools was a natural choice for NETMORPH and therefore easier to implement, faster to simulate, and less memory consuming. However, Cortex3D offers more flexibility to implement user-defined models for various phases of neurodevelopment, and can be used to study many other mechanisms in addition to neurite growth.

#### 3.1.3. Simulation tools for spiking neuronal networks

We present our user experience with three common tools for simulation of large spiking networks of point neuron models. In addition to testing and comparing the simulation tools, we discuss the flexibility of simulation tools to implement user-defined model components. We tested the common tools, NEST (version 2.8.0) with PyNN (version 0.8.0) used as an interface and Brian (version 2.0). All of the tested packages are well documented and additional support is offered through user groups. The general tendency to develop Python based simulation tools or Python interface to simulation tools saves time when analyzing the obtained simulation results, since the same Python modules for analysis and visualization of data can be combined with each simulation tool. Parallelization is supported by NEST and PyNN, however it is still under development in Brian. An earlier version of Brian offers a model fitting method for tuning the statistics of the interspike intervals in spiking neuron models. In Brian version 2.0, this option is under development. NEST and PyNN do not provide direct tools for model fitting. However, both Brian and NEST can easily be combined with external Python modules for model fitting. For fast exploration of models, for example in the early phase of model development, or for incorporating nonstandard biophysical mechanisms in the model, Brian offered more flexibility. In NEST and PyNN, the components of the model have to be either selected from the list of existing models or implemented by extending the simulation tool to include new models. Brian provides more flexible framework for implementation of user-defined models. Model components are specified directly as strings of ODEs. Various models can easily be implemented, however they still rely on the existing functionalities of the simulation tool.

### 3.2. Evaluation and comparison of computational models

#### 3.2.1. Neuronal signal transduction models

Based on our large review of postsynaptic signal transduction models for long-term potentiation and depression (Manninen et al., 2010), we found out that it would have been often time consuming or even impossible to try to reproduce the simulations results. First, not all the details of the models, such as equations, variables, inputs, outputs, compartments, parameters, and initial conditions, were given in the original publications. For example, even just missing to give the inputs in the publications makes the reimplementation and reproduction of the simulation results difficult or impossible with signal transduction models. Second, most of the models were not available in model databases or were not open access, and sometimes even the simulation tool or programming language used was not named in the publications. Third, comparison to previous models was non-existent. Even qualitative comparison was difficult because only a few publications provided graphical illustrations of the model components or the graphical illustrations were misleading by having also components that were not actually modeled. We concluded that the value of computational models for understanding molecular mechanisms of synaptic plasticity would be increasing only with detailed descriptions of the published models and sharing the codes online.

We listed the models we tried to reimplement, resimulate, and compare based on the information in the original publications in Tables 2, 5. In Table 5, we can see that four out of seven models were available in the model repositories but this is because four of the models were chosen to this study because of the availability of the code. Thus, the ratio of models available online is generally not this high. In addition, most of the publications gave all the details of the models as text, tabular format, supplementary material, or at least in the model code. We were able to reproduce Figure 1C of the publication by Delord et al. (2007). From the publication by d'Alcantara et al. (2003), we decided to reproduce only Figures 3D–F. After fixing one mistake in the equations by d'Alcantara et al. (2003), we were able to reproduce most of the simulation results. We were able to reproduce all the other curves, except our maximum value for α-amino-3-hydroxy-5-methylisoxazole-4-propionic acid receptor (AMPAR) activity was about 220 % whereas the original maximum value in Figure 3D was about 280 %. The reason behind the different value might be that not all parameter and initial values were given in the original publication. We were not able to completely implement the model by Zachariou et al. (2013) because not all the information of the model was given in the original publication. More information about the reproducibility issues of the models can be found in our previous publications (Manninen and Linne, 2008; Manninen et al., 2011).

**Table 5:** Evaluation of the neuronal signal transduction models.

**Model**	**Online**	**Language**	**Equations**	**Parameters**	**Init. cond**.	**Repro., Repli**.	**Compa**.
d'Alcantara et al., 2003	No	MATLAB^Ⓡ^	All appendix	Most text	Most appendix, text	++	Tested
Hayer and Bhalla, 2005	DOQCS	GENESIS/Kinetikit, MATLAB^Ⓡ^, SBML	All code, suppl, tab	All code, suppl, tab	All code, suppl, tab	Not tried	Tested
Lindskog et al., 2006	ModelDB	XPPAUT	All code, tab, text	All code, tab	All code	Not tried	Tested
Delord et al., 2007	No	Not given	All text	All text	All text	+++	Tested
Nakano et al., 2010	ModelDB	GENESIS/Kinetikit	All code, suppl, tab, text	All code, suppl, tab	All code, suppl, tab	Not tried	Tested
Kim et al., 2010	ModelDB	XPPAUT	All code, tab, text	All code, tab, text	All code	Not tried	Tested
Zachariou et al., 2013	No	XPPAUT	Most text	Most tab, text	Some text	–	Not tried

***Online:** availability of the model implementation in a model repository by the original authors. **Language/Simulation tool:** programming language or simulation tool used by the original authors to implement the model. **Equations:** availability and format of equations—embedded in the text, appendix, or supplementary material, presented in a table, or described in the publicly available model implementation (code). **Parameters:** availability and format of model parameters (same categories as for Equations). **Init. cond.:** availability and format of initial conditions (same categories as for Equations). **Repro., Repli.:** reproducibility or replicability of the original results with the information given in the original publication. **Compa.:** comparability of the models to each other. We described model implementation in the original publication using the following categories: none, some (at least about one third of the details necessary for model reimplementation is given), most (at least about two thirds are given), or all. Models for which we were not able to reproduce any results are marked by − sign. Models for which we reproduced at least some of the results are marked by one to three + signs depending how well we reproduced the results. We implemented the chosen models with MATLAB^Ⓡ^. See more details in section 3 and in our previous publications (Manninen and Linne, 2008; Manninen et al., 2011)*.

We were the first ones to provide a computational comparison of postsynaptic signal transduction models for synaptic plasticity (Manninen et al., 2011). We evaluated altogether five models, of which two were developed for hippocampal CA1 neurons (d'Alcantara et al., 2003; Kim et al., 2010), two were developed for striatal medium spiny neurons (Lindskog et al., 2006; Nakano et al., 2010), and one was a generic model (Hayer and Bhalla, 2005) (see Tables 2, 5). The model by d'Alcantara et al. (2003), we implemented ourselves in MATLAB^Ⓡ^, but the others we took from model databases. The models by Kim et al. (2010) and Lindskog et al. (2006) we took from ModelDB (Migliore et al., 2003; Hines et al., 2004) in XPPAUT format. The codes were properly commented and clearly written, which made it easy to find the values we wanted to modify. The model by Nakano et al. (2010) we took from ModelDB in GENESIS/Kinetikit format. The model codes were neither intuitive nor commented. However, the database and simulation tool provided helpful explanation files to ease the use of the model files (see more details in Manninen et al., 2011). The model by Hayer and Bhalla (2005) we took from the Database of Quantitative Cellular Signaling (DOQCS, Sivakumaran et al., 2003) in MATLAB^Ⓡ^ format. However, the MATLAB^Ⓡ^ implementation of the model was hard to modify due to issues with parameter handling. Precisely, it was challenging to identify model parameters as the authors opted to hard code numerical values to the MATLAB^Ⓡ^ script instead of using parameter names (see more details in Manninen et al., 2011). We compared the simulation results of the models by using the same input for the models. We ran a set of six simulations with different total concentrations of calcium/calmodulin-dependent protein kinase II and protein phosphatase 1 to see how the behavior of the models changed. Our study showed that when using the same input for all the models, models describing the plasticity phenomenon in the very same neuron type produced partly different responses. On the other hand, the models by d'Alcantara et al. (2003) and Nakano et al. (2010) produced partly similar responses even though they had been built for neurons in different brain areas, and Nakano et al. (2010) did not report using the details of the model by d'Alcantara et al. (2003) when building their model. The models by Lindskog et al. (2006) and Kim et al. (2010) produced also partly similar responses even though they had been built for neurons in different brain areas, but Kim et al. (2010) stated that they used the details of the model by Lindskog et al. (2006) when building their model. Based on these results, we concluded that there is a demand for a general setup to objectively compare the models (see more details in Manninen et al., 2011). In our other study (Manninen and Linne, 2008), we compared the models by d'Alcantara et al. (2003) and Delord et al. (2007) with the same input and the total concentration of AMPARs. We verified that the model by d'Alcantara et al. (2003) was only able to explain the induction of plastic modifications, whereas the model by Delord et al. (2007) was able to explain both induction and maintenance (see also d'Alcantara et al., 2003; Delord et al., 2007).

#### 3.2.2. Astrocyte models

After categorization of astrocyte and neuron-astrocyte models in our previous studies (Manninen et al., 2018a,b), we realized that these models have the same shortcomings as listed in the previous section for neuronal signal transduction models, such as several publications lacked important model details, model codes were rarely available online, graphical illustrations of these models were misleadingly plotting also model components that were not part of the actual model, mathematical equations were sometimes incorrect, and selected model components were not often justified.

**Figure 2:**
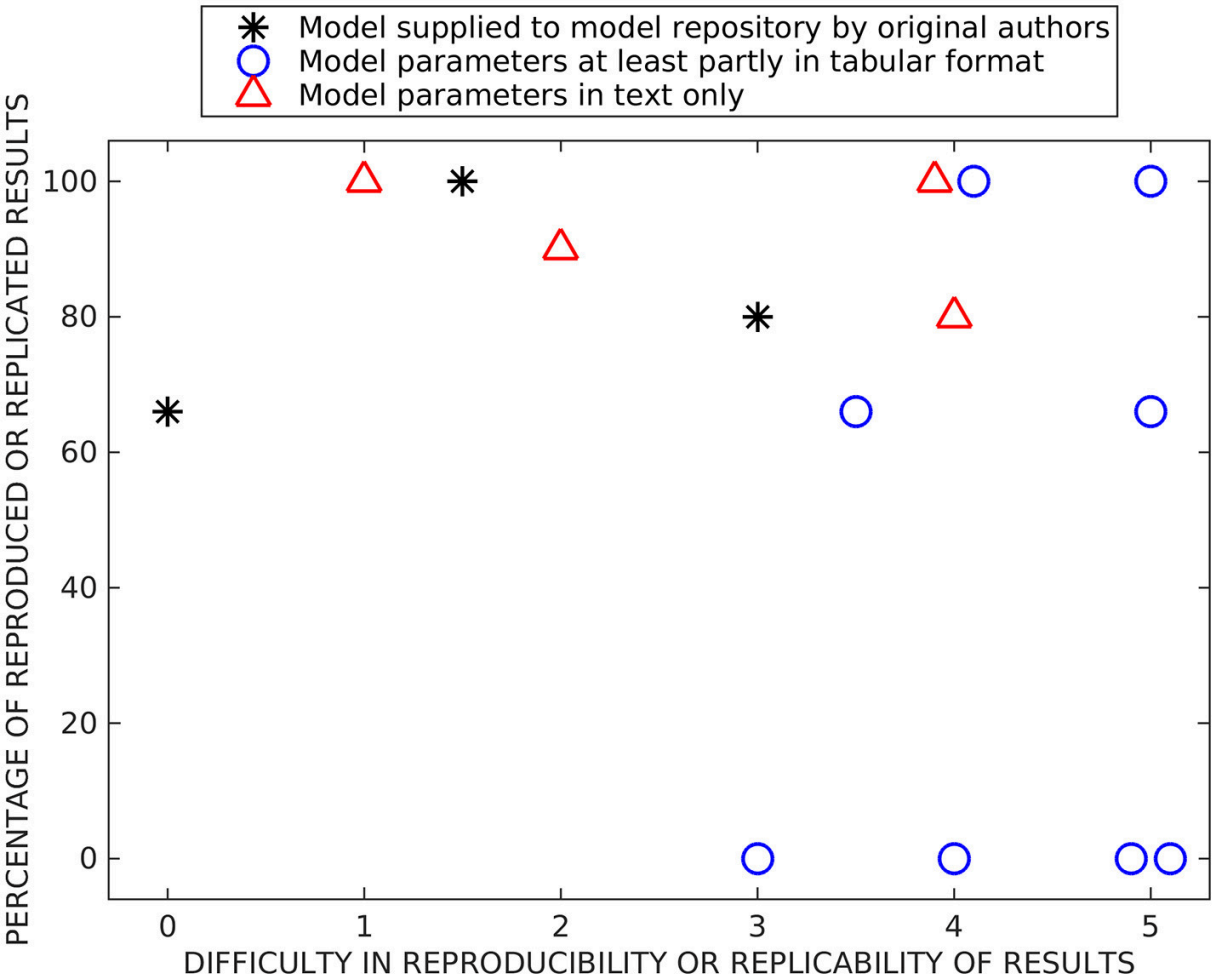
Summary of reproducibility and replicability studies. Both x- and y-values are based on subjective estimation. On the x-axis, we present the difficulty to reimplement, simulate, and reproduce or rerun and replicate previous results (numbers mean the following: 0—immediately, 1—after a few hours of working on the model, 2—after 1 day, 3—after a few days, 4–after 1 week, and 5—after 2 weeks or more). On the y-axis, we present the percentage of reproduced or replicated results. The models are separated into three categories based on were they supplied in model repositories by original authors, were at least part of the parameter values given in a tabular format, and were parameter values given only in text format.

In our previous studies (Manninen et al., 2017, 2018b), we tried to reimplement altogether seven astrocyte models. In the present study, we tried to reimplement two more models. None of the models were available in model repositories by the original authors. However, the model by Lavrentovich and Hemkin (2008) is in ModelDB submitted by someone else (Accession number: 112547). We have provided our implementation for four out of nine models in ModelDB [the models by Lavrentovich and Hemkin (2008), De Pittà et al. (2009), and Riera et al. (2011a,b), and modified version of the model by Dupont et al. (2011), Accession number: 223648]. Most of the publications provided all the details of the models, except the initial conditions, either in text, tabular format, appendix, supplementary material, or in corrigendum. We were able to reproduce all of the chosen original results by Di Garbo et al. (2007) and Lavrentovich and Hemkin (2008) (see Table 6). We reproduced Figures 2, 5, and 8 by Di Garbo et al. (2007) and Figures 3, 4, 5, 7, and 9 by Lavrentovich and Hemkin (2008). We were not able to reproduce any of the important features of the original results by Riera et al. (2011a,b) with the original equations, but after we fixed the found error in one of the equations we were able to reproduce some of the original results in Figure 4B by Riera et al. (2011a) when *X*_IP3_ was 0.43 μM/s between 100 and 900 s and 0 otherwise and all of the original results when *X*_IP3_ was 0.43 μM/s between 100 and 900 s and 0.2 μM/s otherwise. We were able to reproduce most of the original results in Figure 12 by De Pittà et al. (2009). We were able to reproduce well the amplitude modulation but not the frequency modulation part of the figure. Thus the problem might be that the original authors did not provide all the model details correctly for the frequency modulation. We were not able to reproduce any of the important features of the original results in Figures 2 and 3 by Dupont et al. (2011) with the original equations. After we tested our implementation, we realized that there had to be a mistake in the original calcium equation. We tested several different calcium equations based on the equations published by the same authors and were able to reproduce most of the original results with one of the tested equations. At first, we were not able to reproduce Figure 2 by Nadkarni and Jung (2003). After we fixed mistakes in one of the original equations and parameter values, we were able to reproduce most of the original results in Figure 2 by Nadkarni and Jung (2003). Due to several deficiencies in the original model descriptions, we were not able to reproduce the simulation results of the models by Wade et al. (2011, 2012) (see Table 6). In addition, we were not able to completely implement the model by Silchenko and Tass (2008) because not all the information of the model was given in the original publication. More details about the reproducibility issues of the astrocyte models can be found in our previous publications (Manninen et al., 2017, 2018b).

**Table 6:** Evaluation of the astrocyte and neuron-astrocyte models.

**Model**	**Online**	**Language**	**Equations**	**Parameters**	**Init. cond**.	**Repro**.	**Compa**.
Nadkarni and Jung, 2003	No	Not given	All text	All text	No	−/++	Not tried
Di Garbo et al., 2007	No	Not given	All text	All tab	No	+++	Not tried
Silchenko and Tass, 2008	No	Not given	Most appendix, text	Most appendix, tab, text	No	−	Not tried
Lavrentovich and Hemkin, 2008	No (ModelDB by us and others)	Fortran (Python by us, XPP by others)	All text	All corrigendum, text	All text	+++	Tested
De Pittà et al., 2009	No (ModelDB by us)	Not given (Python by us)	All appendix, text	All tab	No	++	Tested
Riera et al., 2011a,b	No (ModelDB by us)	MATLAB^Ⓡ^ (Python by us)	All suppl, tab, text	All suppl, tab, text	No	−/+/+++	Tested
Dupont et al., 2011	No (ModelDB by us)	MATLAB^Ⓡ^ (Mod. model with Python by us)	All text	All tab, text	No	−/++	Tested
Wade et al., 2011	No	MATLAB^Ⓡ^	All text	Most tab, text	Some text	−	Not tried
Wade et al., 2012	No	MATLAB^Ⓡ^	All text	All appendix, tab, text	Most appendix, tab, text	−	Not tried

***Online:** availability of the model implementation in a model repository by the original authors, by us, or by someone else. **Language/Simulation tool:** programming language or simulation tool used by the original authors, by us, or by someone else to implement the model. **Equations:** availability and format of equations—embedded in the text, appendix, supplementary material, or corrigendum, or presented in a table. **Parameters:** availability and format of model parameters (same categories as for Equations). **Init. cond.:** availability and format of initial conditions (same categories as for Equations). **Repro.:** reproducibility of the original results with the information given in the original publication. **Compa.:** comparability of the models to each other. We described model implementation in the original publication using the following categories: none, some (at least about one third of the details necessary for model reimplementation is given), most (at least about two thirds are given), or all. Models for which we were not able to reproduce any results are marked by − sign. Models for which we reproduced at least some of the results are marked by one to three + signs depending how well we reproduced the results. We implemented all the models with Python and/or MATLAB^Ⓡ^, and made some of the models available in ModelDB (Accession number: 223648). We marked the language we used only if we made the model available in ModelDB. See more details in section 3 and in our previous publications (Manninen et al., 2017, 2018b)*.

In addition to testing reproducibility, we also addressed the comparability of the astrocyte models in our previous study (Manninen et al., 2017). We compared the model by Riera et al. (2011a,b) to the model by Lavrentovich and Hemkin (2008), and the model by De Pittà et al. (2009) to our modified version of the model by Dupont et al. (2011). We chose these models because they described similar biological processes. The models by Riera et al. (2011a,b) and Lavrentovich and Hemkin (2008) were spontaneously oscillating models, whereas the other two models used glutamate as stimulus. The overall dynamical behaviors of the models were relatively different. The model by Lavrentovich and Hemkin (2008) oscillated less frequently than the model by Riera et al. (2011a,b). We found out that both models were sensitive to parameter values. Especially, when using the parameter values from the model by Riera et al. (2011a,b) in the model by Lavrentovich and Hemkin (2008), the model by Lavrentovich and Hemkin (2008) behaved differently compared to the behavior with its own parameter values. With a constant glutamate stimulus, the models by De Pittà et al. (2009) and our modified version of the model by Dupont et al. (2011) showed partly similar kind of behavior but there were a few exceptions. First, a higher constant glutamate stimulus value produced higher calcium concentrations with the model by De Pittà et al. (2009) and lower calcium concentrations with our modified version of the model by Dupont et al. (2011). Second, the higher the constant glutamate stimulus value, the faster the model by De Pittà et al. (2009) ceased to oscillate. With pulse wave stimuli, the model by De Pittà et al. (2009) and our modified version of the model by Dupont et al. (2011) produced differing results. In our modified version of the model by Dupont et al. (2011), the calcium concentration oscillated even with the minimum concentration value of the glutamate stimulus pulse. This did not happen with the model by De Pittà et al. (2009). More details about the comparability issues of the astrocyte models can be found in our previous publication (Manninen et al., 2017).

#### 3.2.3. Spiking neuronal network models

We evaluated 10 models listed in Table 4. The majority of the examined publications presented a complete set of equations describing the neuron and synapse models, either in the methods section, appendices, or supplementary material. We found an incomplete set of equations in two of the publications. All model parameters were presented, however not in an easily tractable format. Only one publication presented all the parameters in a tabular format, 6/10 (7/10 if the supplementary material was included) publications partially summarized parameters in a tabular format. None of the publications used the recommendable model description format introduced by Nordlie et al. (2009). Non-systematic model description increases the chance of errors both in the publication and when reimplementing the model. We found several minor errors: wrong naming of parameters, same name used for different parameters in the same article, missing to define some relevant parameters before using them, ambiguities in defining probability distributions used to randomize some of the parameters (e.g., using the wrong name for probability distribution, ambiguity about implementation of probability distribution in the utilized simulation tool), and ambiguities in describing the connectivity scheme. In addition, most of the publications did not give the initial conditions.

Description of network connectivity scheme is equally important part in presentation of network models. The unstructured connectivity is used in 6/10 studies, thus each pair of neurons was connected with equal probability. The other studies included additional connectivity schemes, often distance-dependent connectivity, where the probability of connection decreased with the distance between the pair of neurons, or the small-world connectivity that allows the majority of local and a few long-distance connections. A careful description of the connectivity generating algorithm is advisable for all but the simplest (unstructured) connectivity in order to avoid implementation errors. For example, in one of the publications the authors described network connectivity as “scale-free random” but then assigned a number of outputs to each neuron using a uniform random instead of a power-law distribution. The two studies by Mäki-Marttunen et al. (2013) and Lonardoni et al. (2017) paid additional attention to the generation of connectivity matrix. Both included supplementary material to describe implementations and implications of different connectivity schemes.

The comparison between simulated and experimental data requires extensive data analysis. The lack of standardization of methodology and the ambiguity in presentation of the applied algorithms pose additional obstacles to reproducibility and replicability. All of the models under consideration generated the same type of data, the spontaneous activity exhibiting network-wide bursts, thus the intervals of intensive spiking activity reflecting global synchronization. The analysis of this data often consists of two steps: bursts detection, segmenting the population spike-data into intervals containing bursts, and computing the statistics of different quantitative measures based on the burst detection or on original non-segmented data. Burst detection itself might not be very reliable. A recent review article conducted evaluation of a broad range of burst detection methods and tested them against a carefully crafted benchmark data (Cotterill et al., 2016). The authors concluded that none of the algorithms performed ideally, and suggested a combination of several methods for improving the precision. The issue was not so dramatic in studies that we examined. All of them focused on relatively large bursting events that were easier to identify, compared to the study by Cotterill et al. (2016). Typically, burst detection algorithms depend on free parameters that are manually tuned to the data. However, the fact that methods used in various studies differ and that authors rarely provide the implementation of the algorithms creates an additional obstacle in reproducing the published results. Even bigger variability is presented in selection of methodology to quantify bursting dynamics. The last column in Table 4 illustrates this variability and lists the data measures used in different publications. In the table, burst detection methods are classified into three categories: methods based on spike-data of individual electrodes/neurons, based on population spike-data, and based on global/population firing rate. The measures used to quantify data include analysis of the spike-data statistics, analysis of burst profiles or frequency of their occurrences, frequency analysis, principal component analysis applied to global firing rates, spatial burst propagation, extraction of connectivity from the spike-data of individual neurons, as well as graph theoretic analysis of the extracted connectivity. This lack of standardization in data representation somewhat hinders the comparison between different studies. Reproducibility of the model requires reimplementation of the model equations, burst detection method, and measures used to quantify the data.

The simulation tools range from the custom-made software in MATLAB^Ⓡ^ or C++ to the public simulation tools of spiking neuronal networks (e.g., Brian, NEST, and NEURON). Three out of 10 listed studies provide the full model implementation, namely Masquelier and Deco (2013), Mäki-Marttunen et al. (2013), and Lonardoni et al. (2017). From these studies, we attempted to replicate the results that demonstrate time evolution of model variables and the global dynamical regime of the model, for example adaptation variables, cell membrane potential, and spike raster plots. The replicability of the three studies is summarized in Table 7. The model by Masquelier and Deco (2013) is available in Brian version 1.4.0 and Python version 2.6, and we ran it in Brian version 1.4.1 and Python version 2.7. The code contains model implementation, the list of parameters, and the plotting function sufficient to replicate Figures 4, 5 from the article. The replication of Figure 4, the illustration of neuron and network dynamics, was straightforward. In Figure 5, the neuronal adaptation mechanism was examined and the basic model was tested for two different values of the adaptation time constant τ_*a*_. We replicated the result obtained for τ_*a*_ = 1.6 s but failed to replicate the results for τ_*a*_ = 1.2 s. This might be caused by different versions of Python and Brian used in the original study and in our replicability test. The model by Lonardoni et al. (2017) is available in NEURON/Python format (versions of the software not indicated). It required installation of an additional nonstandard Python package. The model is well documented and supported by many implementation details. The code downloaded from DRYAD repository included model implementation, the code for generating connectivity matrices, as well as three test examples and three examples of connectivity matrices. The first attempt to run the model using Neuron 7.1. produced errors. After contacting the authors, we obtained the correct version for the simulation tool (Neuron 7.3) and Python packages, as well as valuable instructions how to use the code. Under Neuron 7.3, all three test examples worked. We were able to use two out of three connectivity matrices, but not the biggest one (*N* = 4,096 neurons) used in the article. Attempt to run the biggest matrix failed most likely due to memory issues. However, we managed to replicate the rasterplots in Figure 2A by Lonardoni et al. (2017) using a smaller matrix (*N* = 1,024 neurons) and after modifying one parameter. In a smaller network, the burst propagation in Figure 2B by Lonardoni et al. (2017) was somewhat less evident. Thus, we were able to replicate most of the original results by Lonardoni et al. (2017). The study by Mäki-Marttunen et al. (2013) contains two models, a network of HH neurons and a network of leaky-IF (LIF) neurons. We replicated Figure 3 from the article. The first, HH model, is available in MATLAB^Ⓡ^ format (version R2010a/R2011b) using own code and it was possible to replicate it. The second, LIF model, is available in PyNEST format (Python version 2.4.3 and NEST version 2.0.0). The software versions are indicated in the code. We managed to replicate the result using Python version 2.7 and NEST version 2.2.1. Running the same code in a newer version of the simulation tool, NEST version 2.8.0, failed to produce any bursting dynamics. All of the studies provided well-documented models and full set of parameters. However, the replicability of the results was hindered by common problems related to versions of the utilized software. These examples illustrate the need to provide detailed information about simulation environment, in addition to model description and implementation.

**Table 7:** Evaluation of the spiking network models.

**Model**	**Online**	**Language**	**Equations**	**Parameters**	**Init. cond**.	**Repli**.
Latham et al., 2000	No	Not given	All text	All tab, text	No	Not tried
Giugliano et al., 2004	No	Not given	All text	All tab, text	No	Not tried
French and Gruenstein, 2006	No	MATLAB^Ⓡ^	All text	All text	No	Not tried
Gritsun et al., 2010	No	C++, MATLAB^Ⓡ^	Most appendix, text	All tab, text	No	Not tried
Gritsun et al., 2011	No	C++, MATLAB^Ⓡ^	Some text	Some tab, text	No	Not tried
Baltz et al., 2011	No	Brian v2, Python	All text	All text	No	Not tried
Maheswaranathan et al., 2012	No	C++, MATLAB^Ⓡ^	Most text	Most tab, text	No	Not tried
Mäki-Marttunen et al. (2013)	ModelDB	MATLAB^Ⓡ^, NEST	All code, text	All code, tab, text	All code	+++
Masquelier and Deco, 2013	ModelDB	Brian v1, Python	All code, text	All code, tab, text	All code	++
Yamamoto et al., 2016	No	Not given	All text	All text	No	Not tried
Lonardoni et al., 2017	DRYAD	NEURON, Python	All code, suppl, text	All code, suppl, tab, text	All code	++

***Online:** availability of the model implementation in a model repository by the original authors. **Language/Simulation tool:** programming language or simulation tool used by the original authors to implement the model. **Equations:** availability and format of equations—embedded in the text, appendix, or supplementary material, presented in a table, or described in the publicly available model implementation (code). **Parameters:** availability and format of model parameters (same categories as for Equations). **Init. cond.:** availability and format of initial conditions (same categories as for Equations). **Repli.:** replicability of the original results with the information given in the original publication. We described model implementation in the original publication using the following categories: none, some (at least about one third of the details necessary for model reimplementation is given), most (at least about two thirds are given), or all. Models for which we replicated at least some of the results are marked by one to three + signs depending how well we replicated the results*.

#### 3.2.4. Summary of reproducibility and replicability studies

Figure 2 shows our subjective evaluation of the difficulty in timewise to reproduce and replicate the original simulation results and the percentage of reproduced and replicated original results. The list of issues affecting the evaluation of models included: (1) complexity of the reproduced/replicated model, (2) model description in the original publication (in a tabular format, as text, or as a supplementary material, and the amount of details given), (3) possible errors in the model description, (4) report of versions of the simulation tools and packages, (5) person who reimplemented the model, thus the experience of the researcher, and (6) user support from the authors of the model.

We separated all tested reproducibility and replicability models (see Tables 5–7) into three classes according to presentation in related publications: models described fully in the text (all parameters embedded in the text), models with parameters at least partially (and in some cases entirely) given in a tabular format, and the studies which supplied model implementation to the public repositories. We carried out reproducibility studies for the first two categories and replicability studies for the last category. As expected, the replicability studies were less difficult than most of the reproducibility studies, the replication times ranged from working immediately to 2 days. The percentage of replicated results was high in all studies (more than 60 %), and the main obstacle was incompatibility of simulation tool versions. Reproduction time for models described entirely in text ranged from a few hours to a week. Surprisingly, we were able to reproduce on average better the results from these models than the rest of the models, including the three models that we tried to replicate by rerunning the available model implementations (see Figure 2). The reason might be a difference in complexity of models, as these models tended to be simpler than others. The majority of the reproduced models presented most or all the parameter values in a tabular format. The time needed for reproducing these models ranged from a few days to more than 2 weeks. Even though parameter values were given, at least partly, in a tabular format for eight models, we were able to reproduce the original results completely only with two of these models and none of the original results with four of these models. Thus, this category of models had a huge variation in percentage of reproduced results, indicating that some other issues, in addition to model presentation in the article, determined the success of reproduction. The difficulty to reproduce the results increased even when only one parameter value was missing or one mistake in equations. Our results showed that the four models for which we were able to reproduce all the original results gave all the equations and parameter values in the original publication, including corrigenda and supplementary material. However, one of the completely reproduced models had a mistake in one of the equations that we had to fix, for all the other completely reproduced models all the details were given correctly in the publications. Moreover, if all the details of the models were given in the original publication, it did not mean that we were able to reproduce all the results. The reason was that often the models had mistakes in parameter values or equations. We should emphasize that small number of model examples in some categories affected the conclusions (only three replication tests and four tests with models fully described in text are shown).

Figure 2 shows some level of correlation between difficulty and accuracy of reproduction/replication studies: all studies that were done relative fast (up to a few days) achieved relatively high reproduction/replication of the original results. The studies that required more time ranged from no reproduction to perfect reproduction. This also reflects the way how these studies were carried out, some models immediately gave good results while others required long time and numerous tests without guarantee of success. The distribution of values reflecting the success was somewhat bimodal, the reproduction results either failed or succeeded with over 60 % reproduced results. The percentage of replicated results were over 60 % for all models. Finally, the large distribution of precisions for some classes of models indicated that additional issues affect the success of reproduction, particularly the complexity and the accuracy of the model description. This should be emphasized in the light of increasing interest for very complex and biophysically accurate multiscale models. While simpler models provide solid reproducibility in relatively short time, complex models require detailed description of the model, preferably with model implementation made publicly available.

## 4. Discussion

We have continually evaluated computational neuroscience and systems biology software and computational models since 2004 while developing new methods and models for computational neuroscience. In this study, we partly summarized results from our previous studies and partly presented new results. We examined selected simulation tools that are intended for simulation of biochemical reactions and subcellular networks in neuronal and glial cells (see also Pettinen et al., 2005; Manninen et al., 2006c; Mäkiraatikka et al., 2007) and for studying the growth and development of neocortical cultures (see also Mäki-Marttunen et al., 2010; Aćimović et al., 2011) and the dynamics of spiking neuronal networks. We have previously provided an extensive overview of more than hundred computational models for postsynaptic signal transduction pathways in synaptic plasticity (Manninen et al., 2010) and more than hundred computational models for astrocytes and neuron-astrocyte interactions (Manninen et al., 2018a,b), where our purpose was to categorize the models in order to make their similarities and differences more readily apparent. In this study, we provided reproducibility and comparability results for some of these models (see also Manninen et al., 2011, 2017, 2018b) with an aim to present the state-of-the-art in the field and to provide solutions for better reproducibility. Additionally, we provided replicability results for spiking neuronal network models.

Our results show that the different simulation tools we tested were able to provide same simulation results when the same models were implemented in them. On the other hand, it was somewhat difficult to reproduce the original simulated results after reimplementing and simulating the models based on the information in the original publications. We were able to reproduce all the original simulation results of four models out of 12 models we tested. The more complete and correct description the model had in the original publication, the more likely we were able to reimplement the model and reproduce the original results. When the parameter values were in a tabular format, it was much easier to reimplement the model because there was no need to go through the whole article looking for the possible values. Mistyped or missing equations and parameter values in the original publications made the reproducibility of the simulation results most difficult. In our replicability studies, we were able to replicate one study and most of the results from the other two studies. The issues encountered while rerunning the models can be attributed to mismatch in versions of the software used for replication and for original model development. Our experiences emphasize the need to supply not only the model description and implementation but also the details of simulation environment, the versions of the software, and the list of necessary packages. The need for better tracking and documentation of the simulation environment and possible solutions are discussed by Crook et al. (2013).

When developing new simulation tools, a multitude of questions should be asked. Naturally, every simulation tool is limited with the adopted modeling framework but should aim at providing the maximal flexibility within that framework. In that context, the following challenges and questions are relevant:

- How big programming load is needed to implement new biological mechanisms?
- How easy is it to incorporate the model components into the existing models from the literature and public databases?
- Does the simulation tool allow flexible level of details when describing different model components?
- Do the version of the simulation tool and packages needed to run the simulations pose a problem for replicability?

Most of the existing simulation tools of spiking neuronal networks impose strict constraint on selection of model components. Those components are implemented as part of the model source code, and the new ones can be added only through extension of the source code which prevents fast modification of model components. Simulation tools that provide basic mechanisms for model implementation and allow flexible description of details, for example directly implementing the model as either ODEs or SDEs (Brian, XPPAUT), by providing interface for adding new components (NEURON), or by providing unit checks (Brian), offer easier manipulation and modification of the model. For the same reason, the simulation tools of this type allow easier extension and reuse of the published models. Several existing tools support development of multiscale models [MOOSE (Ray et al., 2008), GENESIS, NEURON] or implementation of models using more than one standard simulation tool (MUSIC, Djurfeldt et al., 2010). These tools support models where different mechanisms are represented at different level of details. The different versions of the simulation tools and the packages needed can make replicability problematic. It is very important for the tool developers to take this into account.

Our findings on a specific set of published models for different biological systems stress the importance of a variety of aspects of model development. The following challenges and questions are relevant:

- Has the model been checked carefully in the review process and can it produce the results in the publication?
- Is the quality of the code sufficient?
- Is the model properly validated against experimental wet-lab data and correctly representing the biological findings?
- What new biological, modeling, and computational aspects the model provides on top of the previously published models?
- Are all the details of the model equations and biological components given in the publication in a clear way, preferably in a tabular format?
- Which programming language or simulation tool was used to implement the model, which data analysis methods were used, and are the implementation of the model and data analysis methods available online?

It is important to emphasize that a good-quality model implementation supplied with the original publication improves not only replicability of the study but also understanding of the model itself. This is already important during the review process. Reviewers should have the possibility to rerun the model code, check the simulated results, and compare the simulated and experimental data during the review process (see also Eglen et al., 2017; Rougier et al., 2017). Equally important is to clearly explain what new components the model has in comparison to previously published models and what old and new results the model can show. Verbal description is a suboptimal way to present complex mathematical formalisms and algorithms. It often turns out to be incomplete and a number of ambiguities emerge when attempting to reimplement a model, usually not evident at first. More systematic and compact description of all model details, such as equations, parameter values, initial conditions, and stimuli, using, for example, a tabular format proposed by Nordlie et al. (2009) and Manninen et al. (2017) and a supplementary material presenting a metadata and meta-code are needed for successful reproduction of the published scientific results. Tools to manage, share, and, most importantly, analyze and understand large amounts of both experimental and simulated data are still needed (Bouchard et al., 2016). However, suggestions how to design workflows for electrophysiological data analysis (Denker and Grün, 2016) and how to structure, collect, and distribute metadata of neurophysiological data (Zehl et al., 2016) has already been proposed. Our example of replicability of spiking network models points at a bottleneck in reproducibility and replicability created by the lack of commonly adopted methodology and publicly available code for analysis of simulated data. Following the good practices in development of data-analysis methods, careful description of the methods in the article, and supplying the code with method implementation in addition to the model implementation are necessary steps to ensure model reproducibility and replicability.

Best practices for description of neuronal network models (Nordlie et al., 2009) and minimum information requirements for reproduction (Le Novère et al., 2005; Waltemath et al., 2011a) have been suggested. Moreover, many XML-based model and simulation representation formats, such as SBML (Hucka et al., 2003), CellML (Lloyd et al., 2004), NeuroML (Gleeson et al., 2010), SED-ML (Waltemath et al., 2011b), and LEMS (Cannon et al., 2014), have been developed. On the other hand, Jupyter Notebook (earlier known as IPython Notebook) could be a potential solution to enhance reproducibility and accessibility. In addition to giving all the details needed for model implementation, it is equally important to categorize the biological details of the models, such as neuron models, ion channels, pumps, receptors, signaling pathways, synapse models, in tabular format in publications (see e.g., Manninen et al., 2010, 2018a,b). If not possible to publish via journal due to page limitations, providing the implementation of the model and data-analysis method in a public and widely adopted simulation tool or programming language (e.g., Python) in some of the available model repositories, for example in ModelDB and BioModels database (Le Novère et al., 2006), is a must. Regardless of all the available formats and tools, many authors do not publish their models in a format that is easily exchangeable between different simulation platforms or provide their models at all in model repositories. All these issues should be carefully considered in the training of both experimental and computational neuroscientists (see also Akil et al., 2016).

Throughout this study, we evaluated, reimplemented and reproduced, and replicated a range of models incorporating different levels of biological details and modeling scales. The models and biological mechanisms included some relatively conventional examples but also some that only recently attracted larger attention within the computational community. As the experimental methodology and protocols advance and various neurobiological mechanisms become better understood, the new challenges for computational modeling emerge. Few examples are molecular diffusion in synaptic clefts, dendritic spines, and in other neural compartments (Chay et al., 2016; Hepburn et al., 2017), models of neurodevelopmental phenomena (Tetzlaff et al., 2010; van Ooyen, 2011), or wider range of plasticity mechanisms explored using conventional spiking networks (Miner and Triesch, 2016). Finally, one can aim beyond network modeling formalism and include extracellular space, for example similarly to the approach adopted in the Cortex3D simulation tool (Zubler and Douglas, 2009).

All the above suggestions would greatly improve the replicability and reproducibility of the published results, reduce the time needed to compare the model details and results, and support model reuse in complementary studies or in the studies extending the range of biophysical mechanisms and experimental data.

## Author contributions

TM: Designed the study, acquired and reimplemented the neuronal signal transduction and astrocyte models, simulated the models, evaluated and tabulated the results, and coordinated the production of the final version; JA: Designed the study, evaluated the simulation tools for growth and spiking neuronal networks, acquired and reran the spiking neuronal network models, and evaluated and tabulated the results; RH: Contributed to the design of the study, reimplemented, simulated, and evaluated an astrocyte model, contributed to the testing of the simulation tools for growth, and interpreted biological terminology and knowledge; HT: Contributed to the design of the study, contributed to the interpretation of network growth and activity studies, and interpreted biological terminology; M-LL: Conceived, funded, and designed the study, took part in the selection and evaluation of all models and tools, and interpreted the results in terms of replicability and reproducibility. All contributed to the drafting of the manuscript and approved the final version of the manuscript. All other work reported in the present publication, as motivation for the topic, is cited and is based on the work done previously in Computational Neuroscience Research Group in Tampere, Finland.

### Conflict of interest statement

The authors declare that the research was conducted in the absence of any commercial or financial relationships that could be construed as a potential conflict of interest.
